# Pharmacological interventions targeting cognition and related clinical outcomes in vascular dementia: a systematic review and meta-analysis of randomized controlled trials

**DOI:** 10.1007/s00228-026-04158-9

**Published:** 2026-08-24

**Authors:** Tianyu Cheng, Lin Zhang, Yizhou Zhang, Huazheng Liang, Junyan Wang, Gengzhen Chen, Yujia Huo

**Affiliations:** 1 The People’s Hospital of Chenghai District, Shantou City, Guangdong Province China; 2 The School of Public Health and Preventive Medicine, Monash University, Melbourne, Australia; 3 Suzhou Industrial Park Monash Research Institute of Science and Technology, Monash University, Suzhou, China; 4 Monash University-Southeast University Joint Research Institute (Suzhou), Southeast University, Suzhou, China; 5 State Key Laboratory of Mechanism and Quality of Chinese Medicine, Institute of Chinese Medical Sciences, University of Macau, Macao, SAR China; 6 Centre for Pharmaceutical Regulatory Sciences, University of Macau, Macao, SAR China; 7 Faculty of Health Sciences, University of Macau, Macao, SAR China; 8 Centre for Smart Process Engineering, Great Bay University, Dongguan, 523808 China; 9 Centre for Simulation and Modelling of Particulate Systems, Southeast University-Monash University Joint Research Institute, Suzhou, 215213 China

**Keywords:** Vascular dementia, Pharmacological interventions, Cognitive outcomes, Herbal medicinal products, Systematic review, Meta-analysis, Randomized controlled trials

## Abstract

**Purpose:**

Randomized evidence for pharmacological interventions in vascular dementia (VaD) is fragmented. We conducted a VaD-focused, scale-specific systematic review and meta-analysis.

**Methods:**

We searched four databases for English-language parallel-group randomized controlled trials (1990–2025). Comparable placebo-controlled outcomes were pooled using random-effects meta-analysis; non-poolable outcomes were synthesized according to SWiM. Risk of bias and certainty were assessed with RoB 2 and GRADE.

**Results:**

Sixteen trials included 5,668 participants across trial cohorts; with one exception, all were published in 2012 or earlier. EGb 761^®^ was evaluated in three trials; nimodipine, galantamine, memantine, and rivastigmine in two each; and pentoxifylline, citicoline, sulodexide, donepezil, and Tianzhi granule in one each. Galantamine reduced ADAS-Cog/11 scores (2 trials, *n* = 1,332; MD − 2.01, 95% CI − 3.18 to − 0.85) and ADAS-Cog/13 scores (2 trials, *n* = 1,328; MD − 2.51, 95% CI − 3.87 to − 1.15), and memantine reduced ADAS-Cog/11 scores (2 trials, *n* = 752; MD − 2.20, 95% CI − 3.24 to − 1.15); certainty was moderate. EGb 761^®^ reduced SKT scores (3 trials, *n* = 283; MD − 2.65, 95% CI − 5.17 to − 0.12), but heterogeneity was extreme (I²=92.8%; very low certainty). Galantamine showed no clear NPI benefit (2 trials, *n* = 1,310; MD − 0.13, 95% CI − 4.05 to 3.79), and memantine showed no clear GBS benefit (2 trials, *n* = 595; MD − 1.93, 95% CI − 4.69 to 0.84). Evidence for the other seven interventions was mainly single-trial and non-poolable; functional and global outcomes did not establish consistent benefit.

**Conclusion:**

Galantamine and memantine showed small short-term cognitive effects; EGb 761^®^ suggested a fragile, very low-certainty signal; patient-important benefits remain uncertain.

**Supplementary Information:**

The online version contains supplementary material available at https://doi.org/10.1007/s00228-026-04158-9.

## Introduction

Vascular dementia (VaD) is the most severe clinical expression of vascular cognitive impairment and is attributable to cerebrovascular injury and its downstream neurovascular consequences [1, 2]. Its presentation is heterogeneous, ranging from strategic or multiple infarcts to diffuse small-vessel disease, and often includes executive dysfunction, slowed processing speed, neuropsychiatric symptoms, impaired daily function, and substantial caregiver burden [1–9]. This heterogeneity complicates both trial design and interpretation of treatment effects.

Management of VaD is anchored in vascular risk-factor control, secondary stroke prevention, rehabilitation, and supportive care [1, 6, 10, 11]. No disease-specific pharmacotherapy is approved by major regulators. Cholinesterase inhibitors and memantine are nevertheless used off-label in selected patients, primarily to target cognitive symptoms, although previous reviews have found only small cognitive effects and uncertain functional or global clinical benefit [10, 12–14]. Mechanistic rationales span glutamatergic excitotoxicity, cholinergic deficits, oxidative stress, neuroinflammation, mitochondrial dysfunction, and selected plant-derived pathways [15–22], but mechanistic plausibility is not evidence of clinical benefit. Clinical use also requires attention to contraindications and adverse effects [23–25]. The present review addresses this narrower symptomatic pharmacological question rather than the full multimodal management of VaD.

Interventions evaluated for cognitive and related clinical outcomes include conventional pharmacological agents and selected herbal medicinal products. These were considered within one pharmacological-intervention framework. EGb 761^®^ was classified as a standardized Ginkgo biloba extract, whereas Tianzhi granule was classified as a proprietary Chinese herbal formulation.

Existing syntheses answer related but distinct questions. Battle et al. evaluated cholinesterase inhibitors across VaD and other vascular cognitive impairment populations [12]; McShane et al. reviewed memantine across dementia syndromes [13]; Chan et al. synthesized Chinese herbal medicines using Chinese and international databases [26]; Dang et al. ranked 21 drugs using MMSE, ADL, and adverse-reaction networks [27]; and Masserini et al. mapped and meta-analyzed the broader vascular cognitive impairment literature [28, 29]. A narrower review can still add value by focusing on clinically diagnosed VaD or extractable VaD subgroups, transparently identifying any retained mixed-population evidence, retaining distinct cognitive instruments and scale versions, and pooling only comparable end-of-treatment assessments.

We therefore systematically reviewed parallel-group RCTs of pharmacological interventions targeting cognition and related clinical outcomes in VaD-focused adult trial populations. We aimed to quantify comparable placebo-controlled effects, structure non-poolable evidence without vote counting, evaluate risk of bias and certainty, and clarify whether cognitive changes were accompanied by functional, global clinical, caregiver-reported, or safety outcomes.

## Methods

### Protocol, design and eligibility

This systematic review and meta-analysis was conducted and reported in accordance with PRISMA 2020 [30] and was registered in PROSPERO (CRD420251186730). The original registration combined pharmacological interventions and aerobic exercise with broader mechanistic, pharmacological-exercise synergy, and translational objectives. At an early methodological scoping stage, before data extraction and quantitative synthesis, these broader components were discontinued because they represented clinically and methodologically distinct review questions requiring different eligibility criteria, evidence types, comparators, outcome frameworks, and synthesis methods. The completed review was therefore restricted to parallel-group randomized trials of pharmacological interventions targeting cognition and related clinical outcomes in vascular dementia. This was a substantive post-registration amendment. The PROSPERO version history documents the amended scope, timing, rationale, and implications while preserving the original registration. Aerobic exercise and other non-pharmacological interventions were not systematically evaluated, and no inference regarding their effectiveness should be drawn from this review.

#### Study design

We included randomized controlled trials with a parallel-group design. We excluded observational studies, uncontrolled single-arm/open-label studies, case reports/series, study protocols without results, and reports with insufficient outcome data for extraction.

#### Population

We included adults with clinically diagnosed vascular dementia (VaD), including possible/probable/definite VaD and common subtypes (e.g., subcortical ischemic VaD, small-vessel disease-related VaD, and multi-infarct dementia). Trials enrolling broader dementia populations were eligible when VaD-specific outcome data were separately extractable. One retained exception was GAL-INT-6 [31], which prospectively enrolled participants with probable VaD or Alzheimer’s disease with cerebrovascular disease and reported combined randomized results; this trial was retained as part of the vascular-dementia-focused evidence base but was not described as exclusively VaD and was treated as an applicability limitation. VaD diagnosis otherwise had to be established using recognized clinical criteria with vascular corroboration (e.g., NINDS-AIREN with neuroimaging evidence, DSM-based dementia diagnosis supported by ischemic scores and CT/MRI findings, or ICD-10 criteria corroborated by neuroimaging) [32, 33]. We excluded studies focusing on non-vascular dementias without separable VaD data, as well as cerebrovascular disease or stroke cohorts without a dementia diagnosis.

#### Interventions and comparators

Eligible interventions were pharmacological treatments intended to improve cognition or related clinical outcomes in VaD. They were considered within a single pharmacological-intervention framework comprising conventional pharmacological agents and selected herbal medicinal products. Herbal products were classified according to their specific formulation and clinical identity rather than being collectively categorized as traditional Chinese medicine: EGb 761^®^ was classified as a standardized Ginkgo biloba extract, whereas Tianzhi granule was classified as a proprietary Chinese herbal formulation. Eligible comparators were placebo, no specific anti-dementia treatment, standard care, or an active pharmacological comparator. Active-comparator trials were retained for synthesis without meta-analysis but were not pooled with placebo-controlled comparisons. Non-pharmacological interventions—including exercise, rehabilitation, cognitive training, ADL-oriented programmes, and caregiver-support programmes—together with surgical, device-based, cell, gene, biologic, vaccine, and nanomedicine interventions, were excluded consistently because they did not address the final pharmacological review question.

#### Outcomes

Studies were required to report at least one extractable efficacy outcome. The primary outcome domain was global cognition (e.g., MMSE, ADAS-Cog, and SKT) [34, 35, 36, 37, 38]. The Syndrom-Kurztest (SKT) is a brief performance test of memory and attention in which lower scores indicate better performance [38]. Secondary domains comprised domain-specific cognition, neuropsychiatric symptoms, global clinical status or severity [39, 40, 41, 42, 43, 44, 45], functional status and activities of daily living (ADL/IADL), caregiver-reported impact when available, and safety/tolerability. For each outcome, the randomized treatment-end assessment was prioritized. Rules for selecting change scores, endpoint values, and analysis populations are described in Sect. 3.4.

### Information sources and search strategy

We systematically searched PubMed, Embase, the Cochrane Library, and Web of Science. Google Scholar was used only for supplementary citation cross-checking and was not treated as a separate database source in the PRISMA counts. Controlled vocabulary and free-text terms for VaD and randomized trials were combined with two intervention retrieval blocks: conventional pharmacological agents and herbal medicinal products or selected plant-derived interventions. The blocks were combined with OR to improve retrieval sensitivity and did not represent two separate systems of treatment. Searches covered 1 January 1990 to 31 December 2025; the prespecified lower date limit was selected to encompass contemporary VaD research criteria developed in the early 1990s, including the NINDS-AIREN criteria published in 1993 [32], and the randomized pharmacological trial literature that followed. Eligibility was restricted to English-language publications, without geographical restriction. Full strategies are provided in Supplementary Table 5. Two reviewers independently screened titles/abstracts and full texts; records were excluded at title/abstract screening as clearly irrelevant only when they did not evaluate a treatment intervention in a clinically diagnosed VaD population or were not eligible primary randomized studies. Disagreements were resolved by discussion and, when required, third-reviewer adjudication.

### Data extraction and quality appraisal

Data extraction was performed independently by two researchers, with discrepancies resolved by a third researcher. We collected study characteristics, diagnostic criteria, baseline severity information, intervention and comparator details, treatment duration, and analysis population. For each reported outcome we extracted the outcome domain and instrument, follow-up, data type (change, endpoint, binary, or ordinal), scale direction, group sizes, study-level effect estimate or reported group data, available precision measure, and reporting limitations. For outcomes not included in meta-analysis, we additionally recorded the harmonized direction of the numerical point estimate, the relevant RoB 2 judgment when available, and the reason that pooling was not appropriate (Supplementary Table 3, Sheets A–G).

Clinical and methodological comparability was assessed before quantitative synthesis. Studies were eligible for meta-analysis only when they evaluated the same intervention and comparator, used the same outcome instrument and scale version, and reported sufficiently comparable end-of-treatment assessments. ADAS-Cog/11, ADAS-Cog/13, VADAS-Cog, and SKT were therefore retained as distinct outcomes. Numerical data required for meta-analysis included randomized or analyzed sample sizes, means, standard deviations, and confidence intervals for intervention and placebo groups. Outcomes that did not meet these criteria were retained in a structured synthesis without meta-analysis rather than excluded from the review.

Risk of bias was assessed using Cochrane RoB 2 [46]. Outcome-specific assessments were completed for prespecified primary outcomes and outcomes contributing to meta-analysis. Each result was judged across bias arising from randomization, deviations from intended interventions, missing outcome data, outcome measurement, and selection of the reported result, with overall judgments of low risk, some concerns, or high risk. When reporting was insufficient, judgments followed the RoB 2 algorithm. Figure 2 presents results contributing to meta-analysis, and Supplementary Table 2 presents assessments for all prespecified primary outcomes. Secondary outcomes in the SWiM table that were not separately assessed are explicitly labelled as such rather than assigned an inferred judgment.

Certainty of evidence for outcomes included in meta-analysis was assessed with GRADE [47] across risk of bias, inconsistency, indirectness, imprecision, and publication bias/other considerations. Certainty was rated as high, moderate, low, or very low. Summary of Findings tables are provided in Fig. 6 and Supplementary Tables 6, and full five-domain evidence profiles with judgment rationales are provided in Supplementary Table 7.

### Data analysis

Pairwise meta-analyses were conducted only when at least two placebo-controlled RCTs evaluated the same intervention, comparator, instrument and scale version, and a comparable end-of-treatment assessment. Network meta-analysis was not undertaken because the evidence network was sparse, interventions were poorly connected, and outcome instruments and reporting metrics were heterogeneous. All outcomes included in the meta-analyses used change-from-baseline data; change scores and endpoint absolute values were not mixed within a pooled analysis. Continuous effects were summari, zed as mean differences (MDs) with 95% confidence intervals (CIs) using inverse-variance random-effects models, with between-study variance estimated by restricted maximum likelihood (REML) [48, 49]. Binary outcomes were to be summarized as odds ratios when sufficiently comparable. Heterogeneity was evaluated with Cochran’s Q and I². Leave-one-out analyses were performed for meta-analyses with at least three studies, and fixed-effect models were examined when heterogeneity was low. Change-score SDs were extracted directly when reported. Where mean changes were reported with standard errors, SDs were calculated as SD = SE × sqrt(n); where 95% CIs for a group mean change were reported, the corresponding SE was derived using the appropriate critical value and converted to an SD. For Kanowski et al. (1997), the change-score SD was calculated from baseline and week-24 SDs as SDchange = sqrt(SDbaseline² + SDfollow-up² − 2r × SDbaseline × SDfollow-up), assuming *r* = 0.5. Sensitivity analyses used *r* = 0.3 and 0.7. No meta-analysis included at least 10 studies; funnel plots and Egger’s tests were therefore not performed. Analyses used R 4.5.2, meta 8.2.1 (including metacont()), and readxl 1.4.5.

For outcomes not amenable to meta-analysis, synthesis without meta-analysis followed the SWiM reporting guideline [50]. Studies were grouped first by outcome domain and then by intervention, comparator type, instrument, and end-of-treatment follow-up. Placebo and active-comparator trials were interpreted separately. Original measurement scales were retained, and effect directions were harmonized according to the numerical point estimate so that ‘favors intervention’ or ‘favors comparator’ did not depend on statistical significance. Intention-to-treat analyses, between-group comparisons, prespecified outcomes, and end-of-treatment assessments were prioritized when multiple analyses were available. Structured tables report study-level estimates, precision, sample size, analysis population, effect direction, RoB 2 judgment when available, and reason not pooled. Narrative conclusions considered magnitude, direction, precision, risk of bias, consistency, and completeness of reporting rather than counting statistically significant findings. Because most intervention-outcome combinations were represented by single heterogeneous trials, these results were treated as descriptive and hypothesis-generating. A completed SWiM reporting checklist is provided in Supplementary Table 8.

## Results

### Study selection

**Fig. 1:**
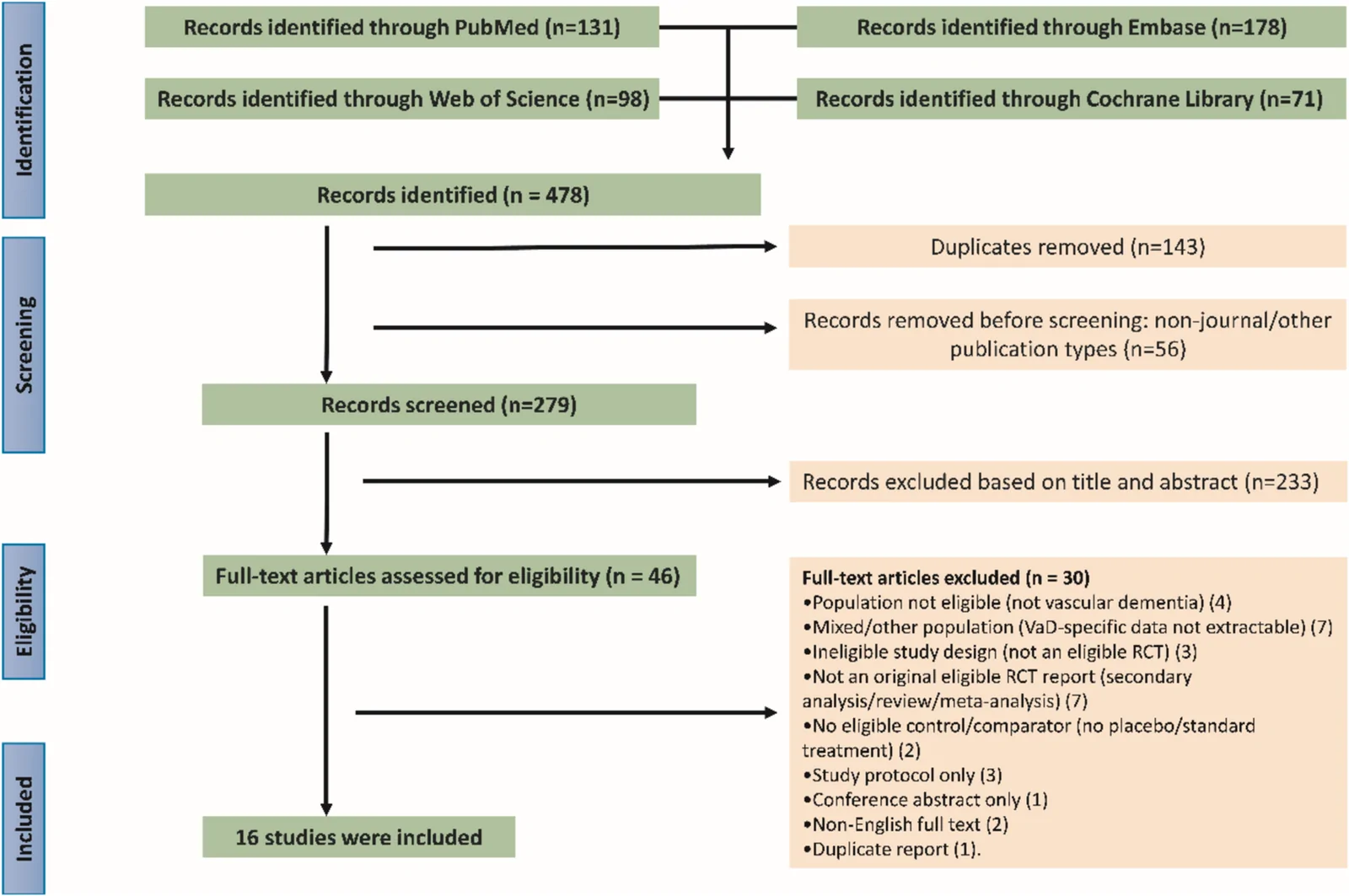
Flowchart of the search and study selection process

Searches of PubMed, Embase, the Cochrane Library, and Web of Science identified 478 records (Fig. 1; Supplementary Table 5). After duplicate and clearly non-journal records were removed, 279 unique records underwent title/abstract screening. A further 233 were excluded as clearly irrelevant because they did not evaluate a treatment intervention in a clinically diagnosed VaD population or were not eligible primary randomized studies, leaving 46 full texts. Thirty full texts were excluded for ineligible population (*n* = 4), mixed populations without extractable VaD data (*n* = 7), ineligible RCT design (*n* = 3), secondary analysis or review/meta-analysis (*n* = 7), no eligible comparator (*n* = 2), protocol without results (*n* = 3), conference abstract only (*n* = 1), duplicate report (*n* = 1), or non-English full text (*n* = 2). Sixteen studies were included. Quantitative synthesis was restricted to sufficiently comparable comparisons; all other eligible results were retained in the structured synthesis without meta-analysis.

### Characteristics of included studies

Sixteen parallel-group RCTs enrolled 5,668 participants across the included trial cohorts, with 3,208 allocated to an active pharmacological intervention and 2,460 to placebo or an active/standard-therapy comparator; trial sample sizes ranged from 30 to 974. VaD subgroup data were used where separately available. The interventions comprised eight conventional pharmacological agents and two herbal medicinal products: EGb 761^®^, a standardized Ginkgo biloba extract, and Tianzhi granule, a proprietary Chinese herbal formulation.

EGb 761^®^ was evaluated in three trials [51–53]; nimodipine [54, 55], galantamine [31, 56], memantine [57, 58], and rivastigmine [59, 60] were each evaluated in two trials; and pentoxifylline [61], citicoline [62], sulodexide [63], donepezil [64], and Tianzhi granule (TZ) [65] were each evaluated in one trial. The included studies were published between 1996 and 2020, with most conducted during 1997–2007. Most trials were undertaken in Europe (*n* = 10), with additional studies from North America (*n* = 1) and Asia (*n* = 2), and three multinational, cross-continental trials spanning multiple regions. Treatment duration ranged from 22 to 52 weeks. Age eligibility was variably reported: three trials specified a minimum age threshold (at least 50 years in two trials [51, 53] and at least 55 years in one trial [52]), whereas others reported mean and SD without explicit age , limits. Across trials, the broad age span was 35–94 years, and baseline mean ages in intervention and control groups generally clustered around 64–78 years with minimal between-group differences (Table 1).

**Table 1:** Characteristics of included studies. Age is reported as mean ± SD (range), unless otherwise stated; age data for Román et al. (2010) are reported as mean ± SE, as presented in the original publication, and the trial’s overall eligibility range was 35–94 years. Abbreviations: SD, standard deviation; SE, standard error; BID, twice daily; TID, three times daily; QD, once daily; PO, oral administration (per os); EGb 761^®^, standardized Ginkgo biloba extract; TZ, Tianzhi granule; EPMID, European Pentoxifylline Multi-Infarct Dementia (study acronym); GAL-INT-6, galantamine international trial 6; mg, milligram; g, gram

Study characteristics						Intervention			Control		
First author	Year	Country	Institution	Overall dementia type	Intervention	Patient number	Age	Regimen/Dosing	Patient number	Age	Regimen/Dosing
EPMID Study Group	1996	Multinational (Europe)	Multicenter EPMID Study Network	Multi-infarct dementia	Pentoxifylline	148	70.0 ± 9.3 (range 45–90)	Pentoxifylline 400 mg TID for 9 months	141	69.4 ± 9.0 (range 46–87)	Matching placebo, TID for 9 months
Auchus AP	2007	Multinational (Europe, North America, Israel, Asia, Australia)	University of Tennessee Health Science Center and Memphis VA Medical Center	Vascular dementia	Galantamine	396	72.3 ± 9.0	Flexible-dose galantamine: start 4 mg BID for 4 weeks -> 8 mg BID for 4 weeks -> up-titrate to 12 mg BID as tolerated (maintenance 16–24 mg/day; mean ~ 16.4 mg/day, ~ 20.2 mg/day during maintenance)	390	72.2 ± 8.8	Matching placebo, identical BID titration schedule
Cohen RA	2003	USA	Brown Medical School; University of Iowa	Vascular dementia	Citicoline	15	78.1 ± 5.8	Citicoline 500 mg BID for 12 months	15	78.0 ± 5.1	Placebo BID for 12 months
Erkinjuntti T	2002	Multinational (10 countries)	Helsinki University Central Hospital; GAL-INT-6 Study Group	Vascular dementia; Alzheimer’s disease with cerebrovascular disease	Galantamine	396	75.0 ± 6.84	Galantamine: 24 mg/day PO BID; 1-week titration 4 -> 24 mg/day for 6 months	196	75.2 ± 7.32	Matching placebo, BID for 6 months
Napryeyenko O	2009	Multicenter (Ukraine)	Outpatient clinics: 16 psychiatric or neurological hospitals	Vascular dementia; Alzheimer’s disease with cerebrovascular disease	Ginkgo biloba extract EGb 761^®^	200	Mean mid 65 (subgroup means 63–66)	EGb 761^®^ 240 mg/day (120 mg PO BID) for 22 weeks	200	Mean mid 65 (subgroup means 63–64)	Matching placebo, 2/day for 22 weeks
Ihl R	2012	Single country (Ukraine)	Outpatient clinics: neurological/psychiatric hospitals in Ukraine	Vascular dementia; Alzheimer’s disease with cerebrovascular disease	Ginkgo biloba extract EGb 761^®^	202	Mean mid 65 (subgroup means 64.9–65.8)	EGb 761^®^ 240 mg PO QD for 24 weeks	202	Mean mid 65 (subgroup means 64.2–66.5)	Matching placebo, PO QD for 24 weeks
Kanowski S	1997	Germany	Outpatient neurology/psychiatry practices in Berlin, Freiburg and Karlsruhe; Free University Berlin and associated centers	Multi-infarct dementia; Alzheimer’s disease	Ginkgo biloba special extract EGb 761	79	Mean mid 70 (male 69.7 ± 11.2; female 70.2 ± 9.4)	EGb 761 240 mg/day PO (120 mg BID) for 24 weeks after 4-week placebo run-in	77	Mean mid 70 (male 66.4 ± 11.5; female 72.0 ± 9.6)	Matching placebo, BID for 24 weeks after 4-week placebo run-in
Mok V	2007	Hong Kong (China)	Prince of Wales Hospital, The Chinese University of Hong Kong	Subcortical vascular dementia	Rivastigmine	20	75.7 ± 5.1	Rivastigmine 1.5 mg BID -> titrated to 3 mg BID (6 mg/day) over 4 weeks; maintained 26 weeks	20	74.1 ± 6.6	Matching placebo, BID for 26 weeks
Moretti R	2003	Italy	Department of Clinical Medicine and Neurology, University of Trieste	Subcortical vascular dementia	Rivastigmine	104	75.67 ± 2.98	Rivastigmine 3 mg/day -> titrated to 6 mg/day after 12 weeks; continued for 12 months	104	75.67 ± 2.98	Aspirin 100 mg/day for 12 months
Orgogozo J-M	2002	France, Belgium, Switzerland	48 centers (France); 1 center (Belgium); 1 center (Switzerland); CHU Pellegrin, Hôpital Broca, and participating hospitals	Vascular dementia	Memantine	165	76.6 ± 6.5	Memantine 20 mg/day PO (titrated: 5 mg week 1 -> 10 mg week 2 -> 15 mg week 3 -> 20 mg/day from week 4 to week 28)	156	76.1 ± 6.86	Matching placebo, identical titration schedule for 28 weeks
Pantoni L.	2000	Finland, Denmark, Sweden	Multicenter (Helsinki University Central Hospital; Kuopio University Central Hospital; Piteå Hospital; Sahlgrenska University Hospital, etc.)	Multi-infarct dementia	Nimodipine	128	73.7 ± 7.18	Nimodipine: 30 mg TID for 26 weeks	131	74.9 ± 6.43	Placebo TID for 26 weeks
Pantoni L.	2005	Multicenter (Italy, Spain, others per trial)	University of Florence; Hospital Severo Ochoa, Madrid; Bayer Medical Department, Italy; OPIS Data, Italy	Subcortical vascular dementia	Nimodipine	124	75.2 ± 6.1	Nimodipine: 30 mg TID (90 mg/day) for 52 weeks	118	75.4 ± 6.0	Matching placebo, identical schedule for 52 weeks
Parnetti L.	1997	Italy	Multicenter Italian geriatric centers (Perugia University; Milano IRCCS Maggiore Hospital; Chieti University; Geneva University; Bologna Malpighi Hospital)	Vascular dementia	Sulodexide	46	Mean mid 75 (range 65–80)	Sulodexide 50 mg PO BID for 6 months	40	Mean mid 76 (range 66–80)	Pentoxifylline 400 mg PO TID for 6 months
Román GC.	2010	Multinational (9 countries: USA, Canada, Europe, etc.)	Multicenter academic and research institutions (e.g., University of Texas Health Science Center at San Antonio; Brown Alpert Medical School; Sunnybrook Health Sciences Centre; UC Davis; San Francisco VA Medical Center)	Vascular dementia	Donepezil	648	73.4 ± 0.4 (SE)	Donepezil 5 mg PO QD for 24 weeks	326	72.3 ± 0.5 (SE)	Matching placebo, PO QD for 24 weeks
Shi J.	2020	China	Department of Neurology, Dongzhimen Hospital, Beijing University of Chinese Medicine (multicenter study, 16 centers in China)	Vascular dementia	Tianzhi granule (TZ)	242	64.7 ± 9.2	Tianzhi granule 5 g per sachet, 1 sachet PO TID + placebo donepezil for 24 weeks (after 2-week placebo run-in)	60	64.0 ± 9.2	Placebo matching Tianzhi granule (5 g TID) + placebo donepezil QD for 24 weeks
Wilcock G.	2002	United Kingdom	Bristol University, Department of Care of the Elderly, Frenchay Hospital, Bristol (multicenter; 54 centers in the United Kingdom)	Vascular dementia	Memantine	295	77.2 ± 6.9	Memantine PO, titrated from 5 mg QD with weekly 5 mg increments to 20 mg/day by week 4, then maintained at 10 mg BID (20 mg/day) for 28 weeks	284	77.6 ± 7.0	Matching placebo, identical titration schedule and maintenance (20 mg/day equivalent) for 28 weeks

Baseline disease severity was reported using non-equivalent criteria and instruments. Where explicitly classified, participants generally had mild-to-moderate dementia. For example, the donepezil trial enrolled a predominantly mild VaD population with a mean baseline MMSE of approximately 23.5 [64], whereas the Kanowski trial enrolled mild-to-moderate cases, of whom approximately 79% were classified as mild and 21% as moderate [52]. Inconsistent definitions and incomplete reporting precluded a reliable severity-stratified meta-analysis.

Four trials enrolled mixed diagnostic cohorts. Separately reported VaD subgroup results were used for the three EGb 761^®^ trials [51, 52, 53]. GAL-INT-6 [31] reported combined randomized results for probable VaD and Alzheimer’s disease with cerebrovascular disease and therefore contributed less population-specific evidence than the other trials.

Global cognition was most often measured with the MMSE (11 trials) and the ADAS family (6 trials); other measures included SKT, SCAG, and DRS. Trials also reported domain-specific cognition, neuropsychiatric symptoms, global clinical status, functional/ADL outcomes, caregiver-reported distress, and safety, although the instruments and completeness of reporting varied substantially (Supplementary Tables 1 and 3).

### Risk of bias assessment

**Fig. 2:**
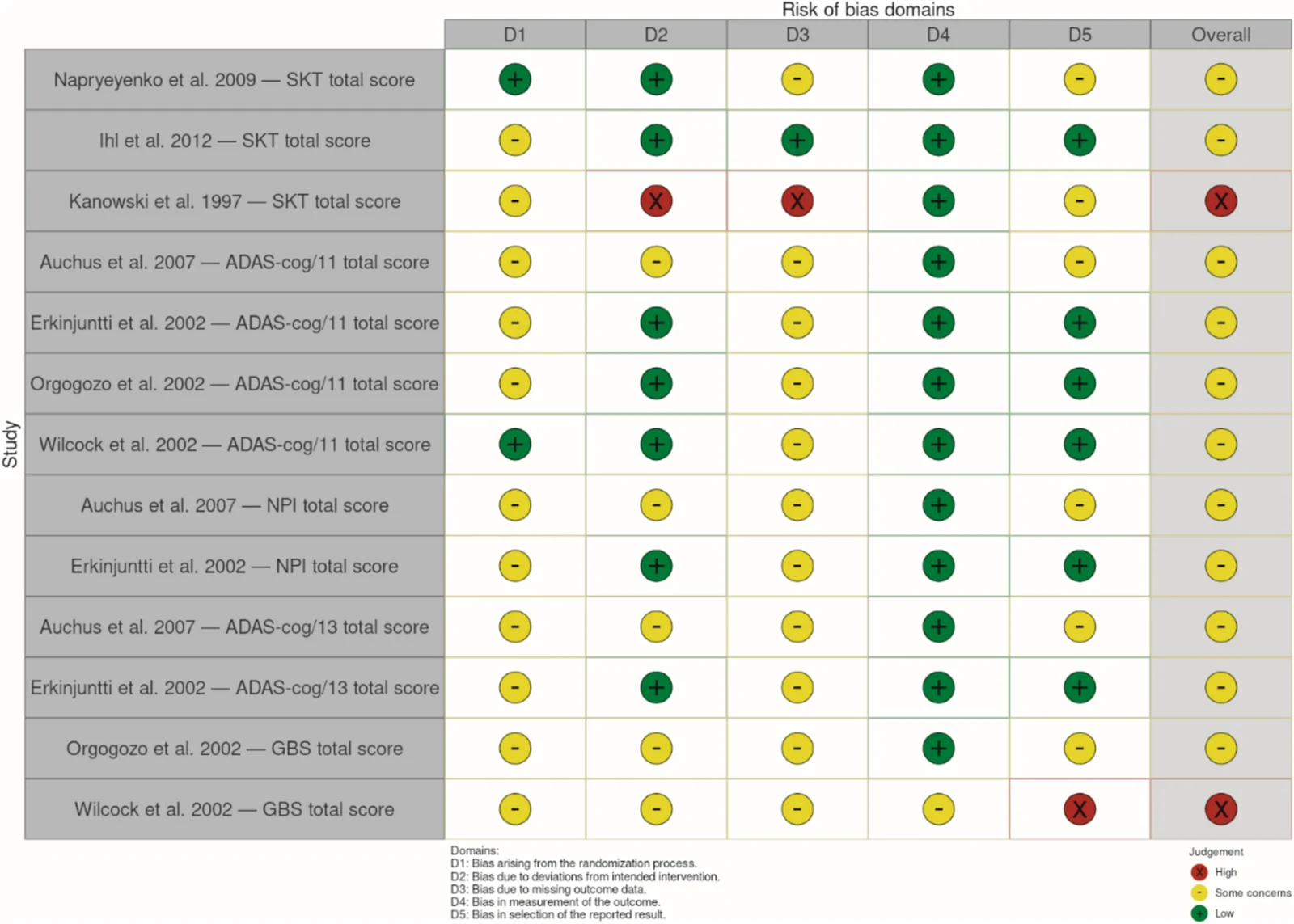
Risk of bias assessment (RoB 2). Traffic-light plot of RoB 2 judgments by domain (D1–D5) and overall (green=low, yellow=some concerns, red=high). Assessments shown are limited to outcomes pooled in the meta-analysis; other main outcomes are reported in Supplementary Table 2. The figure was generated using the robvis web application (https://mcguinlu.shinyapps.io/robvis/)

Because RoB 2 [46] is outcome-specific, we assessed risk of bias for the prespecified primary outcomes and for outcomes contributing to meta-analyses, to support the main quantitative conclusions. For the outcomes included in meta-analysis (Fig. 2), 11/13 outcome assessments were judged as some concerns and 2/13 as high risk (Kanowski et al. 1997-SKT total score; Wilcock et al. 2002-GBS total score). Concerns most frequently related to the randomization process (D1), missing outcome data (D3), and selection of the reported result (D5), while outcome measurement (D4) was generally judged at low risk. Across all primary outcomes (Supplementary Table 2), most assessments were rated as some concerns (24/30), with the remainder rated as high risk (6/30); high-risk judgments were mainly driven by missing outcome data and/or selective reporting.

Traffic-light plot of RoB 2 judgments by domain (D1–D5) and overall (green = low, yellow=some concerns, red=high). Assessments shown are limited to outcomes pooled in the meta-analysis; other main outcomes are reported in Supplementary Table 2. The figure was generated using the robvis web application (https://mcguinlu.shinyapps.io/robvis/).

### Primary outcome (global cognition)

#### ADAS-Cog family (ADAS-Cog/11, ADAS-Cog/13, and VADAS-Cog)

Six randomized trials reported ADAS-Cog-family outcomes at end of treatment (approximately 24–28 weeks). Lower scores indicate better cognition. Four placebo-controlled trials (two galantamine and two memantine) were sufficiently comparable for pairwise meta-analysis; ADAS-Cog/11 and ADAS-Cog/13 were analyzed separately, and VADAS-Cog was retained as a distinct composite outcome (Supplementary Table 3, SheetA).

**Fig. 3:**
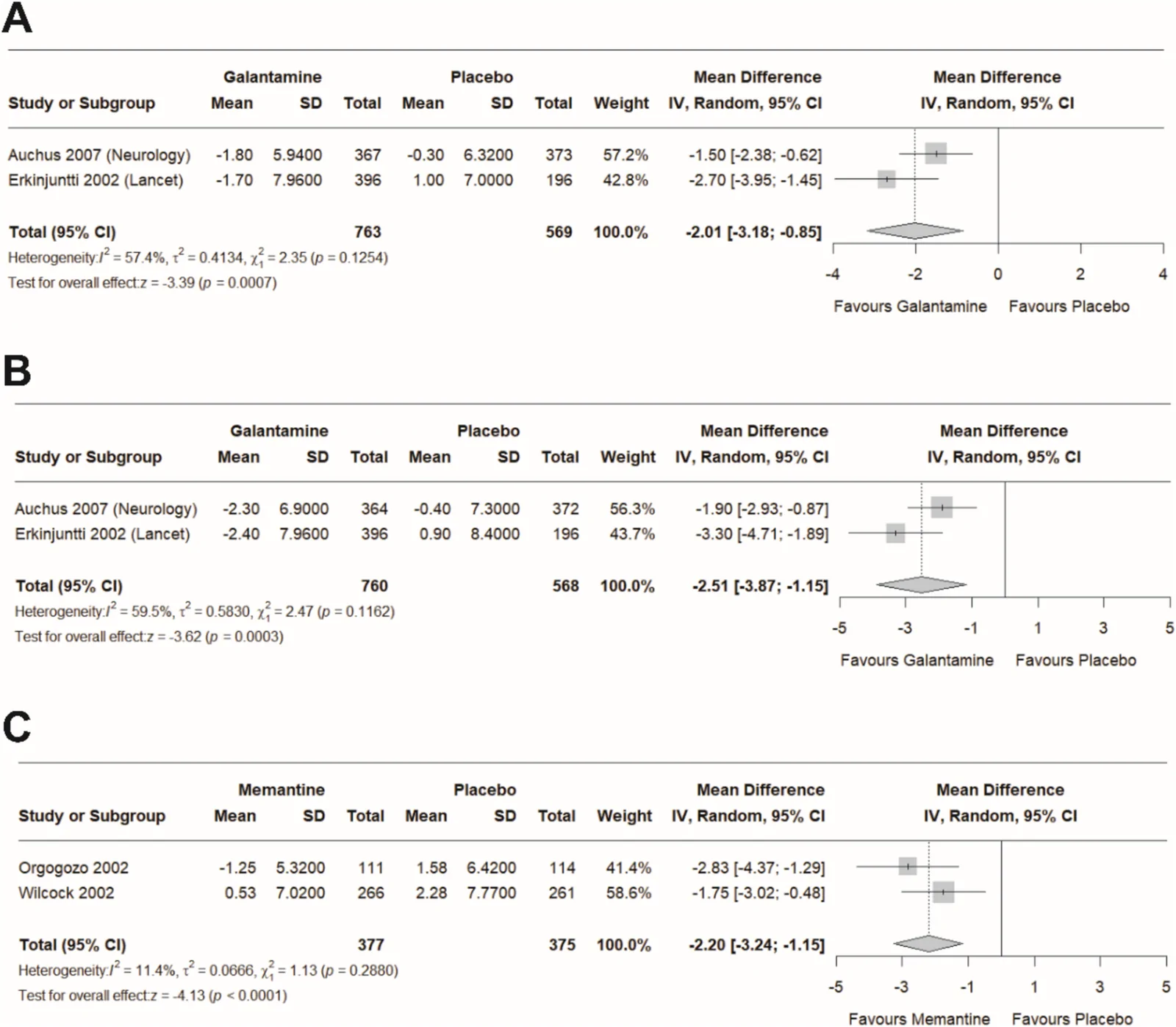
Effects of galantamine and memantine on global cognition (ADAS-Cog) in vascular dementia. Forest plots showing change-from-baseline ADAS-Cog outcomes at end of treatment (~24–28 weeks) for placebo-controlled trials. **A** Galantamine vs placebo on ADAS-Cog/11; **B** galantamine vs placebo on ADAS-Cog/13; **C** memantine vs placebo on ADAS-Cog/11. Pooled effects are mean differences (MD) with 95% confidence intervals using an inverse-variance random-effects model. Lower ADAS-Cog scores indicate better cognition; negative MDs favor the active intervention. Extracted numerical data are provided in Supplementary Table 4A.

Galantamine showed a statistically significant benefit versus placebo on both ADAS-Cog/11 (random-effects MD -2.01, 95% CI -3.18 to -0.85; I²=57.4%) and ADAS-Cog/13 (MD -2.51, 95% CI -3.87 to -1.15; I²=59.5%) (Fig. 3A and B). Memantine also demonstrated a significant improvement versus placebo on ADAS-Cog/11 (MD -2.20, 95% CI -3.24 to -1.15; I²=11.4%) (Fig. 3C).

Two additional trials were retained in the structured synthesis because their outcome definitions or reporting metrics were not compatible with the pooled comparisons. In the donepezil trial, VADAS-Cog favored donepezil (LS mean difference − 1.156, 95% CI -1.98 to -0.33; *p* < 0.01), and ADAS-Cog/11 showed a smaller estimate in the same direction (difference − 0.707, 95% CI -1.40 to -0.02; *p* = 0.0464). In the three-arm Shi trial, VADAS-Cog changes favored Tianzhi granule and donepezil over placebo, with pairwise differences of -2.73 (95% CI -4.58 to -0.88) and − 3.05 (95% CI -4.91 to -1.20), respectively. Each comparison was supported by a single trial (Supplementary Table 3, SheetA).

#### Syndrom-Kurztest (SKT) total score

**Fig. 4:**
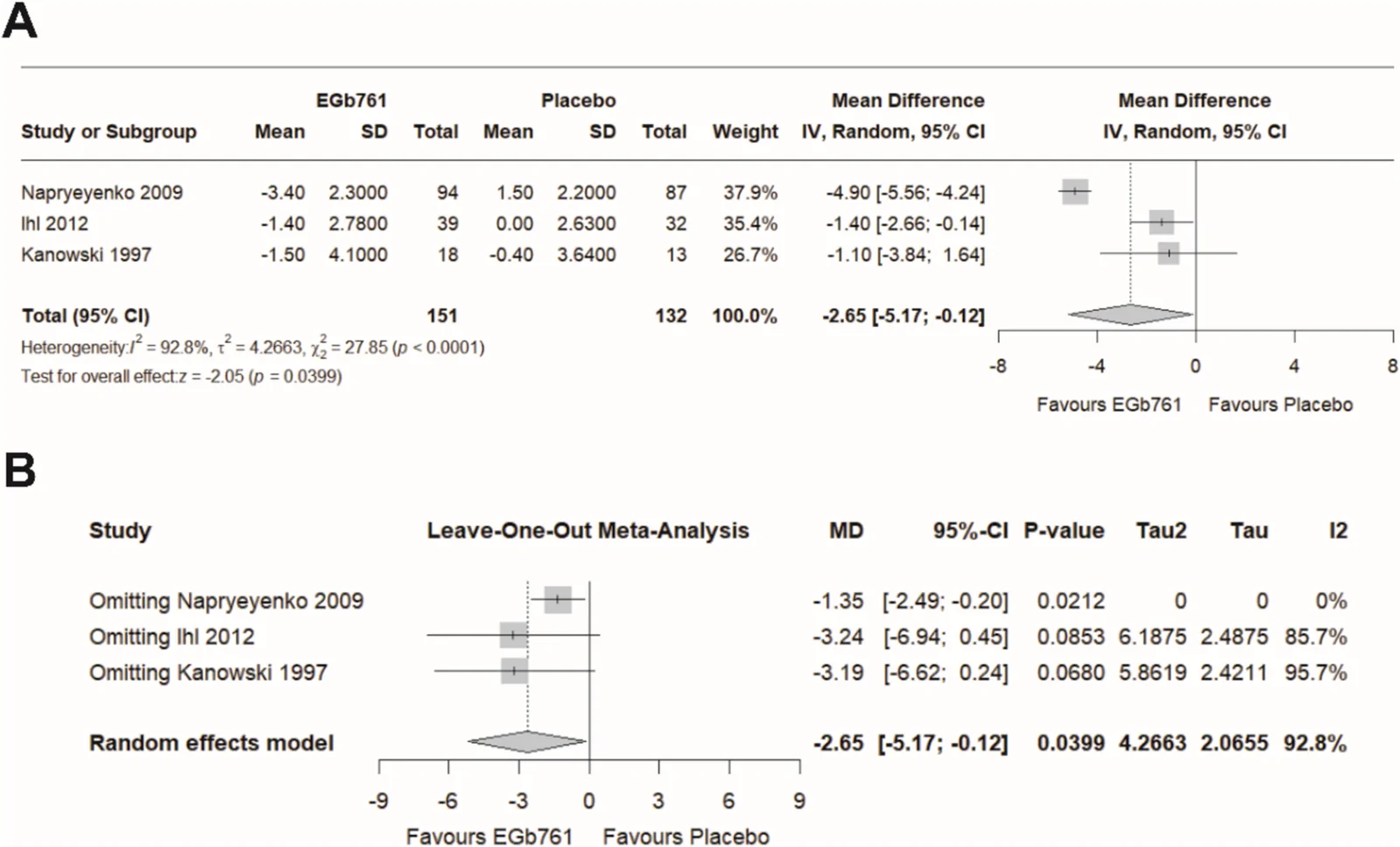
Effects of EGb 761® on cognition (SKT total score) in vascular dementia. **A** Forest plot of change-from-baseline SKT total score at end of treatment for placebo-controlled trials in vascular dementia subgroups. Pooled effects are mean differences (MD) with 95% confidence intervals using an inverse-variance random-effects model. Lower SKT scores indicate better cognition; negative MDs favor EGb 761®. **B** Leave-one-out sensitivity analysis showing the influence of omitting each study on the pooled estimate and heterogeneity. Extracted numerical data are provided in Supplementary Table 4A

Three placebo-controlled randomized trials of Ginkgo biloba extract EGb 761^®^ reported change from baseline in SKT total score in the VaD subgroup. A random-effects meta-analysis showed a mean difference of -2.65 (95% CI -5.17 to -0.12; *p* = 0.0399) in favor of EGb 761^®^ (Fig. 4A), but between-study heterogeneity was extreme (I²=92.8%; τ²=4.27). Leave-one-out analyses showed that the statistical conclusion depended on which study was omitted (Fig. 4B): excluding Napryeyenko 2009 yielded MD -1.35 (95% CI -2.49 to -0.20; I²=0%), whereas excluding Ihl 2012 or Kanowski 1997 yielded MD -3.24 (95% CI -6.94 to 0.45) and MD -3.19 (95% CI -6.62 to 0.24), respectively. Sensitivity analyses varying the assumed pre-post correlation used to derive the Kanowski change-score SD produced similar pooled estimates (*r* = 0.3: MD -2.69, 95% CI -5.25 to -0.13, I²=92.5%; *r* = 0.5: MD -2.65, 95% CI -5.17 to -0.12, I²=92.8%; *r* = 0.7: MD -2.59, 95% CI -5.08 to -0.10, I²=93.5%, Supplementary Table 4B). Thus, the result was not materially altered by the correlation assumption but remained highly inconsistent and sensitive to individual studies.

#### Mini-mental state examination (MMSE)

MMSE was reported across eight interventions, but an overall effect was not estimated because interventions, comparator types, follow-up, analysis populations, and reporting metrics differed (Supplementary Table 3, SheetB). Original-scale study-level estimates and available precision measures were therefore retained.

The donepezil trial reported a 0.472-point difference versus placebo at week 24 (95% CI 0.05 to 0.89). In the three-arm Shi trial, mean changes were + 2.19 with Tianzhi granule and + 2.61 with donepezil versus + 1.35 with placebo (overall three-arm *p* = 0.025); exact ITT pairwise precision was not reported. The two memantine trials produced estimates in different directions: Orgogozo reported changes of + 1.75 versus + 0.52 (*p* = 0.0121), whereas Wilcock reported + 0.24 versus + 0.51 without a between-group precision estimate. Nimodipine, rivastigmine, pentoxifylline, citicoline, and sulodexide comparisons were single-trial, active-comparator, incompletely reported, or analytically incompatible. These estimates do not support a robust comparative conclusion across interventions.

#### Other global cognition

Other global cognitive outcomes could not be pooled because instruments were non-equivalent (Supplementary Table 3, SheetC). Pentoxifylline was associated with a -1.3-point change on the SCAG cognitive-function subscale versus − 0.4 with placebo (*p* = 0.007). In the citicoline trial, DRS changes were − 6.0 (SD 21.2) versus − 5.5 (SD 14.2), but a between-group precision measure was not reported. Both comparisons were supported by single trials and were interpreted descriptively.

### Secondary outcomes

#### Domain-specific cognitive tests

Domain-specific cognitive results were not pooled because instruments, scale directions, follow-up, and reporting formats differed (Supplementary Table 3, SheetD). In VaD subgroups, EGb 761^®^ estimates favored the intervention for category fluency and clock drawing in the Napryeyenko trial (both *p* < 0.01) and for category fluency in the Ihl trial (mean change + 0.8 vs. -0.3; *p* = 0.004). Donepezil favored the intervention on the Number Cancellation Test (difference − 0.131, 95% CI -0.26 to -0.01; *p* = 0.0396) but produced imprecise estimates on CLOX-1, CLOX-2, and EXIT25; galantamine yielded a -1.0-point greater change on EXIT-25 (*p* = 0.041). Nimodipine, rivastigmine, Tianzhi granule, sulodexide, pentoxifylline, and citicoline results varied by instrument and analysis, and several relied on active comparators, within-group tests, post hoc analyses, or unavailable between-group precision. The domain-level evidence was therefore descriptive and did not establish a consistent intervention-specific pattern.

#### Neuropsychiatric symptoms

**Fig. 5:**
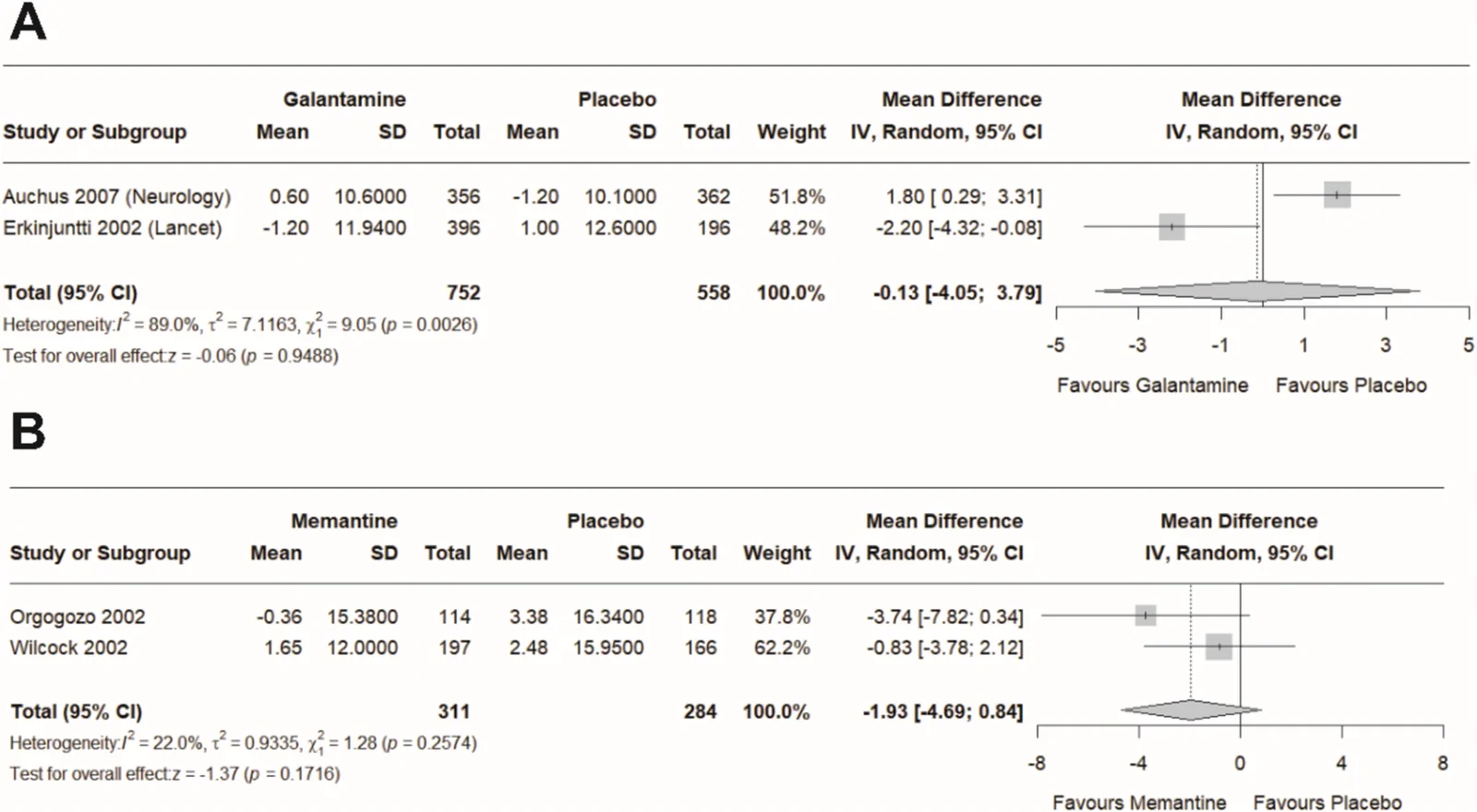
Effects of galantamine and memantine on neuropsychiatric and global clinical outcomes in vascular dementia. Forest plots of change-from-baseline outcomes at end of treatment for placebo-controlled trials. **A** Galantamine vs placebo on Neuropsychiatric Inventory (NPI) total score. **B** Memantine vs placebo on Gottfries–Bråne–Steen Scale (GBS) total score. Pooled effects are mean differences (MD) with 95% confidence intervals using an inverse-variance random-effects model. Lower scores indicate fewer symptoms/less severity; negative MDs favor the active intervention. Extracted numerical data are provided in supplementary table 4A

Neuropsychiatric outcomes were reported with non-equivalent instruments and were retained as study-level estimates (Supplementary Table 3, SheetE). The pooled galantamine NPI estimate was − 0.13 (95% CI -4.05 to 3.79; I²=89%), with individual trial estimates in opposite directions (Fig. 5A). In an EGb 761^®^ VaD subgroup, NPI change was − 4.5 versus − 1.3 (*p* = 0.036), while the responder estimate was 54% versus 31% (*p* = 0.092). The Shi trial reported changes of -3.03 with Tianzhi granule and − 2.21 with donepezil versus − 0.36 with placebo (pairwise *p* < 0.001 and *p* = 0.013, respectively). Caregiver-distress estimates also favored EGb 761^®^ in the Napryeyenko (-5.2 vs. + 0.8; *p* < 0.01) and Ihl (-2.0 vs. + 0.1; *p* = 0.012) VaD subgroups, but each was a single-trial comparison. Emotional or mood results reported with GBS, NOSGER, and GDS were heterogeneous and sometimes limited to active comparators, within-group tests, or unavailable numerical estimates, precluding an overall neuropsychiatric conclusion.

#### Global clinical outcomes

**Fig. 6:**
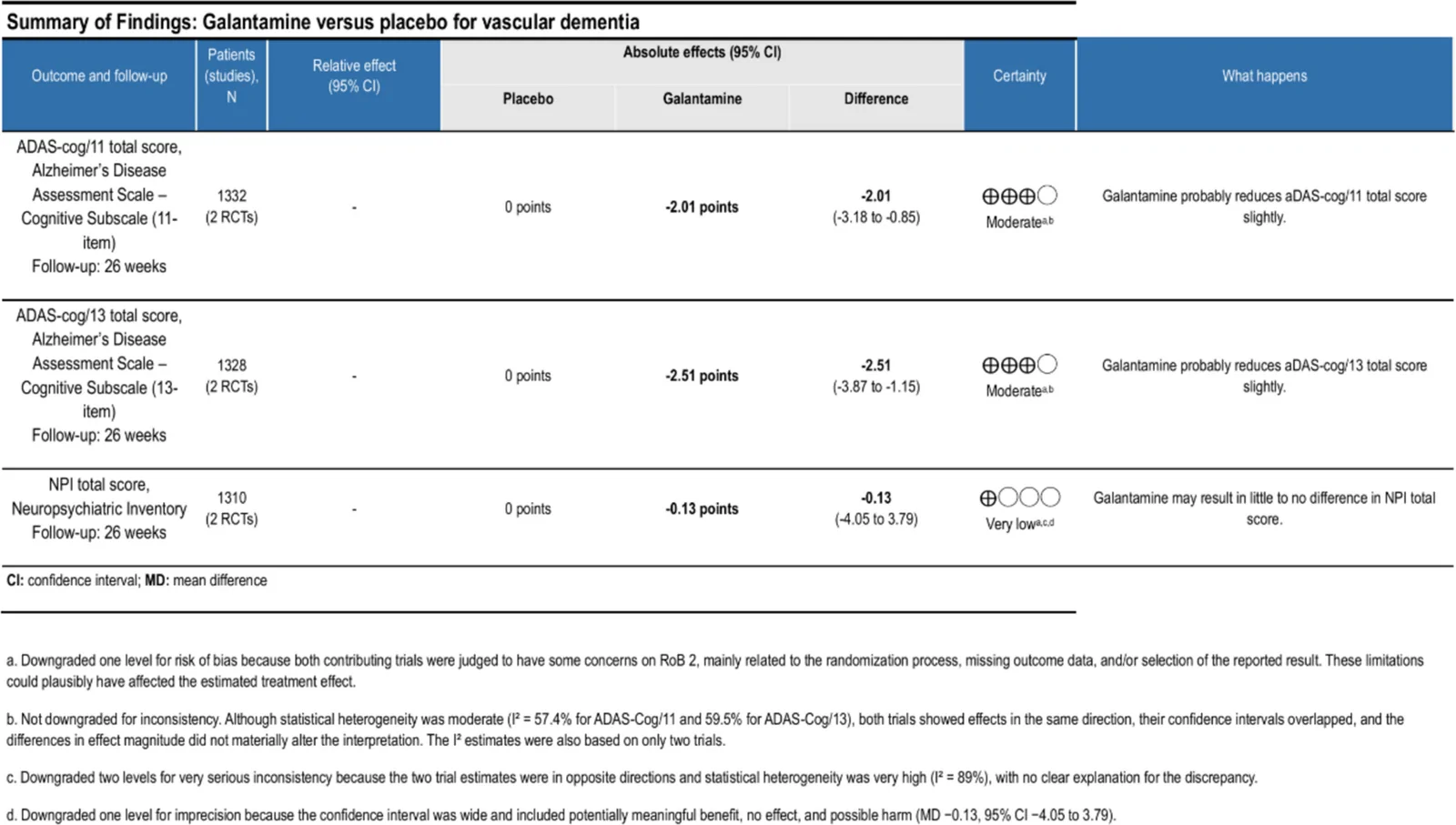
Summary of Findings (GRADE) for galantamine versus placebo in the included vascular-dementia-focused trial populations. Summary of Findings table presenting effect estimates and certainty ratings for galantamine outcomes included in meta-analysis. The figure was generated using GRADEpro GDT (https://gdt.gradepro.org/). Additional Summary of Findings tables are provided in Supplementary Table 6, and full five-domain GRADE evidence profiles are provided in Supplementary Table 7

Global clinical outcomes were measured with non-equivalent instruments and were therefore synthesized as study-level estimates (Supplementary Table 3, SheetF). Memantine showed no clear pooled difference on GBS total score (MD -1.93, 95% CI -4.69 to 0.84; I²=22%; Fig. 5B). Single trials reported estimates favoring pentoxifylline on GBS total (-3.5 vs. -0.7; *p* = 0.028) and EGb 761^®^ in a VaD subgroup (-10.8 vs. + 4.8; *p* < 0.01). CIBIC-plus estimates favored galantamine in Erkinjuntti (74% improved or unchanged vs. 59%; *p* = 0.001) and Tianzhi granule in Shi (improved/stable/deteriorated 73.71%/21.98%/4.31% vs. 58.19%/20.00%/21.82%; *p* = 0.004), whereas the second galantamine trial lacked a precision estimate. Donepezil did not show a clear endpoint difference on CIBIC-Plus (*p* = 0.23). CGI/CGIC, CDR/CDR-SB, and SCAG estimates varied in magnitude, direction, and reporting completeness; consequently, no global clinical measure supported a robust cross-intervention comparison (Fig. 6).

#### Functional outcomes and caregiver-reported impact

Functional outcomes were reported with DAD, ADCS-ADL, ADL/IADL indices, GBS-ADL, ADL-IS, NAB, NOSGER dimensions, and related scales and were not pooled (Supplementary Table 3, SheetF). Galantamine produced a 4.6-point DAD treatment effect in Erkinjuntti (*p* = 0.0017), whereas ADCS-ADL changes in Auchus were 0.7 versus 1.3 (*p* = 0.783). For donepezil, the prioritized trial-end DAD estimate was 1.768 points (95% CI -0.05 to 3.59; *p* = 0.0591), although a week-24 analysis favored donepezil. EGb 761^®^ estimates favored the intervention on GBS-ADL and ADL-IS in two VaD subgroups, while the Kanowski VaD/MID subgroup NAB change numerically favored placebo and lacked a between-group precision estimate. Rivastigmine, nimodipine, memantine, Tianzhi granule, and donepezil comparisons were small, single-trial, imprecise, or inconsistent across instruments. Together with the sparse caregiver-distress results in SheetE, the evidence was insufficient to establish that cognitive changes translated into consistent everyday functional benefit.

#### Safety and tolerability

Safety outcomes were not pooled because adverse-event definitions, denominators, follow-up, and comparative measures were heterogeneous (Supplementary Table 3, SheetG). Twelve trials reported participants with at least one adverse event, eight reported serious adverse events, 10 reported withdrawals due to adverse events, and 10 reported deaths. Gastrointestinal events and treatment discontinuation were prominent in several cholinesterase-inhibitor trials; dizziness, constipation, or confusion were commonly reported with memantine; and nimodipine reports included vascular and neurological events in populations with substantial baseline vascular risk. EGb 761^®^ and Tianzhi granule reports generally described non-serious events but used inconsistent ascertainment. Because most reports did not provide comparable effect estimates, the available safety data do not support a reliable cross-intervention ranking.

No meta-analysis included 10 or more studies; funnel plots and Egger’s tests were therefore not performed.

### Certainty of evidence

Summary of Findings table presenting effect estimates and certainty ratings for galantamine outcomes included in meta-analysis. The figure was generated using GRADEpro GDT (https://gdt.gradepro.org/). Additional Summary of Findings tables are provided in Supplementary Tables 6, and full five-domain GRADE evidence profiles are provided in Supplementary Table 7.

GRADE was applied to quantitatively synthesized outcomes for galantamine, memantine, and EGb 761^®^, with certainty ranging from very low to moderate. For galantamine, ADAS-Cog/11 and ADAS-Cog/13 were of moderate certainty, downgraded one level for risk of bias. Inconsistency was not downgraded because both trial estimates favored galantamine, their confidence intervals overlapped, and the moderate I² estimates (57.4% and 59.5%) were based on only two trials and did not materially alter interpretation. Galantamine NPI evidence was very low certainty because of risk of bias, very serious inconsistency, and imprecision. Memantine ADAS-Cog/11 evidence was moderate certainty, downgraded for risk of bias, while GBS total evidence was low certainty because of risk of bias and imprecision. EGb 761^®^ SKT evidence was very low certainty because of risk of bias, very serious inconsistency, and imprecision. Full judgments and rationales are presented in Supplementary Tables 6 and 7.

## Discussion

### Summary of main findings

This review included 16 parallel-group RCTs of eight conventional pharmacological agents and two herbal medicinal products in VaD-focused trial populations. Quantitative evidence was confined to a few placebo-controlled comparisons. Galantamine and memantine produced small short-term improvements on ADAS-Cog outcomes with moderate certainty, whereas the EGb 761^®^ SKT estimate was of very low certainty because of risk of bias, extreme inconsistency, and imprecision. Most other intervention-outcome combinations were represented by single or analytically incompatible trials. Functional, global clinical, caregiver-reported, and safety results were too sparse or heterogeneous to establish robust cross-intervention conclusions.

### Comparison with previous reviews and incremental contribution

The pooled efficacy estimates were confirmatory. Our galantamine ADAS-Cog/11 result (MD -2.01, 95% CI -3.18 to -0.85) was identical to the pairwise estimate in Battle et al. [12]. Our memantine ADAS-Cog/11 estimate (MD -2.20, 95% CI -3.24 to -1.15) closely matched the Cochrane estimate of -2.15 (95% CI -3.25 to -1.05) [13] and was directionally consistent with the standardized cognitive effect reported by Masserini et al. (Cohen’s d 0.33, 95% CI 0.11 to 0.55) [29]. For Ginkgo biloba, Masserini et al. analyzed the same three SKT trials and reported an unstandardized improvement of 2.56 points (95% CI -0.17 to 5.29; very low certainty) [29], broadly similar in magnitude to our 2.65-point estimate after harmonizing scale direction, but with a confidence interval that crossed the null. The different inferential result despite the same small evidence base reinforces the fragility of the EGb 761^®^ signal. Detailed comparisons are provided in Supplementary Table 9.

The principal contribution of this review is methodological and interpretive rather than the discovery of a previously unrecognized effective treatment. Beyond reproducing previously reported pooled estimates, it provides a broad study-level map of the full range of cognitive outcomes reported across the eligible VaD-focused randomized evidence, including outcomes that could not be meta-analysed, while preserving non-equivalent instruments and presenting their magnitude, direction, precision, and methodological limitations within a structured SWiM framework. The same framework integrates non-poolable functional, global clinical, caregiver-reported, and safety outcomes, clarifying whether cognitive changes were accompanied by patient-important benefit. Unlike broad VCI reviews [28, 29], network rankings based mainly on MMSE and ADL [27], or comprehensive Chinese herbal medicine reviews [26], we focused on clinically diagnosed VaD and separately extractable VaD subgroups, while transparently retaining one combined probable-VaD/AD-with-cerebrovascular-disease trial [31]. We retained ADAS-Cog/11, ADAS-Cog/13, VADAS-Cog, and SKT as distinct outcomes and pooled only the same intervention, comparator, instrument, and comparable treatment-end assessment. This narrower framework preserves scale-level interpretability and shows how little evidence remains directly poolable after clinically and analytically strict eligibility criteria are applied.

Clinical interpretation must remain conservative. A VaD-specific minimal important difference for ADAS-Cog has not been established. A commonly cited threshold of approximately 3 points was derived for clinically relevant worsening in mild Alzheimer’s disease, not for improvement or between-group differences in VaD [66]. The approximately 2-point galantamine and memantine effects are therefore statistically detectable but cannot be assumed to be perceptible to patients or caregivers; Battle et al. likewise judged effects of this magnitude unlikely to be clinically important [12]. Earlier VaD meta-analysis also identified small cognitive effects without consistent behavioral or functional benefit [14], so the cognition-function dissociation observed here confirms an established pattern rather than constituting a new finding.

### Functional outcomes, practice effects, and applicability

The structured functional synthesis supports this cautious interpretation. Some single trials favored galantamine or EGb 761^®^ on particular ADL instruments, but donepezil did not show a clear DAD difference at the prespecified endpoint, and results varied across non-equivalent scales. Caregiver-distress data were limited to two EGb 761^®^ subgroup reports. Baseline severity was also inconsistently characterized; where explicit, populations were mainly mild to moderate, and severity-stratified meta-analysis was not reliable. Consequently, the results should not be generalized confidently to severe VaD or used to identify a severity-defined responder subgroup.

Repeated cognitive testing can produce practice effects, which are recognized confounders in longitudinal cognitive assessment [67]. Concurrent randomized placebo comparisons should reduce bias from practice effects shared by both groups, so practice alone does not explain a between-group mean difference. However, it can obscure underlying decline, complicate within-group interpretations, and interact with attrition, analysis populations, test familiarity, and ceiling effects. This is particularly relevant to single-trial narrative findings and to older trials that did not report alternate forms or procedures intended to limit retest effects. Future VaD trials should prespecify strategies to minimize or model practice effects.

Applicability is further limited by intervention and comparator heterogeneity. EGb 761^®^ is a standardized Ginkgo biloba extract rather than a proprietary Chinese herbal medicine, and Tianzhi granule was the only included proprietary Chinese herbal formulation. The present English-language evidence therefore cannot be generalized to traditional Chinese medicine as a whole. Active-comparator trials, including rivastigmine versus aspirin and sulodexide versus pentoxifylline, do not establish absolute efficacy because the comparator may not be inert. The Tianzhi three-arm trial included placebo and donepezil, but comparable changes versus donepezil should not be interpreted as formal non-inferiority without a prespecified non-inferiority margin and analysis.

### Limitations and certainty of evidence

Several limitations affect certainty. Trials used evolving diagnostic criteria, incompletely reported VaD subtypes and severity, short treatment periods, and predominantly older designs. Excluding mixed-dementia studies without extractable VaD data improved population specificity but reduced generalizability because mixed vascular and neurodegenerative pathology is common in practice. In addition, GAL-INT-6 [31] reported combined results for probable VaD and Alzheimer’s disease with cerebrovascular disease; retaining this trial preserved a key randomized comparison but reduced population specificity for the galantamine estimate. Restriction to English-language publications and omission of Chinese databases may have introduced language and selection bias, particularly for herbal formulations studied in China [26, 68]. Publication bias could not be formally examined because every meta-analysis contained fewer than 10 studies. The sparse, disconnected network and incompatible outcomes precluded network meta-analysis. Risk-of-bias concerns, imprecision, and heterogeneity further limit confidence, especially for EGb 761^®^. Exercise, rehabilitation, cognitive training, ADL-oriented programmes, and caregiver-support interventions were outside the final PICO and were not systematically evaluated; this review therefore cannot inform their effectiveness, although they remain relevant components of comprehensive VaD care.

Synthesis without meta-analysis also has inherent limitations: non-equivalent instruments, incompatible reporting formats, incomplete precision measures, and mostly single-trial intervention-outcome comparisons prevented pooled magnitudes for many outcomes. Effect-direction summaries cannot fully account for effect size or sample size and should be interpreted descriptively. Although the SWiM structure improves transparency, it cannot overcome limitations in the underlying reports.

### Implications for practice and future research

Current guidance appropriately keeps vascular risk-factor management, secondary stroke prevention, rehabilitation, and supportive care at the center of VaD management, while allowing cautious consideration of cholinesterase inhibitors in selected patients [10]. The present results do not support stronger or broader pharmacological recommendations. If a symptomatic agent is considered, expectations should be limited to a possible small short-term cognitive effect, with attention to tolerability and to whether any patient-important functional or global benefit emerges. Evidence for EGb 761^®^ and Tianzhi granule is insufficient to recommend them as representatives of herbal medicine or traditional Chinese medicine generally. Strategic-infarct, multi-infarct, and small-vessel/subcortical vascular syndromes can differ in dominant cognitive deficits, progression, and plausible treatment targets; subtype-defined enrolment is therefore needed to reduce clinical heterogeneity and permit assessment of treatment-effect modification. Future RCTs should also report baseline severity consistently; use vascular-sensitive cognitive measures alongside ADL/IADL, global, quality-of-life, and caregiver outcomes; minimize or model practice effects; prespecify missing-data handling; maintain standardized background vascular care; and provide longer follow-up. Adequately connected, harmonized trial networks would be required before comparative ranking by network meta-analysis becomes credible.

## Conclusion

By separating non-equivalent cognitive instruments and scale versions and integrating non-poolable functional, global clinical, caregiver-reported, and safety outcomes under SWiM, this review clarifies the limited comparability and uncertain clinical translation of the VaD-focused randomized evidence. Across 16 RCTs, reproducible quantitative evidence was limited to small short-term cognitive effects for galantamine and memantine, with uncertain translation into daily function or global clinical benefit. The EGb 761^®^ SKT estimate was highly heterogeneous, sensitive to individual studies, and of very low certainty. Evidence for other conventional agents and for Tianzhi granule was sparse or non-poolable, and safety data did not permit reliable ranking. These findings support cautious interpretation rather than broad treatment recommendations and highlight the need for subtype-defined populations, harmonized vascular-sensitive and functional outcomes, rigorous missing-data and practice-effect methods, and longer follow-up.

## Electronic Supplementary Material

Below is the link to the electronic supplementary material.

Supplementary file 1

Supplementary file 2

Supplementary file 3

Supplementary file 4

Supplementary file 5

Supplementary file 6

Supplementary file 7

Supplementary file 8

Supplementary file 9

## Data Availability

The datasets generated and/or analysed during the current study are available from the corresponding author on reasonable request. The analytic code used in this study is available from the corresponding author on reasonable request.
