# Supplementary material for: Pharmacological interventions targeting cognition and related clinical outcomes in vascular dementia: a systematic review and meta-analysis of randomized controlled trials
Source: Eur J Clin Pharmacol. 2026 Aug 24;82(9):240. doi: 10.1007/s00228-026-04158-9 (PMC13503495; doi:10.1007/s00228-026-04158-9)
Supplement: Supplementary file 1 [file 228_2026_4158_MOESM5_ESM.docx]

**Question: Galantamine compared with placebo in the included vascular-dementia-focused trial populations**

| **Certainty assessment** | | | | | | | **№ of patients** | | **Effect** | | **Certainty** |
| --- | --- | --- | --- | --- | --- | --- | --- | --- | --- | --- | --- |
| **№ of studies** | **Study design** | **Risk of bias** | **Inconsistency** | **Indirectness** | **Imprecision** | **Other considerations** | **Galantamine** | **Placebo** | **Relative (95% CI)** | **Absolute (95% CI)** |  |
| **ADAS-Cog/11 total score (follow-up: 26 weeks; assessed with: Alzheimer’s Disease Assessment Scale – Cognitive Subscale (11-item))** | | | | | | | | | | | |
| 2 | randomized trials | serious^a^ | not serious^b^ | not serious | not serious | none | 763 | 569 | - | MD **2.01 points lower** (3.18 lower to 0.85 lower) | ⨁⨁⨁◯ Moderate^a,b^ |
| **ADAS-Cog/13 total score (follow-up: 26 weeks; assessed with: Alzheimer’s Disease Assessment Scale – Cognitive Subscale (13-item))** | | | | | | | | | | | |
| 2 | randomized trials | serious^a^ | not serious^b^ | not serious | not serious | none | 760 | 568 | - | MD **2.51 points lower** (3.87 lower to 1.15 lower) | ⨁⨁⨁◯ Moderate^a,b^ |
| **NPI total score (follow-up: 26 weeks; assessed with: Neuropsychiatric Inventory)** | | | | | | | | | | | |
| 2 | randomized trials | serious^a^ | very serious^c^ | not serious | serious^d^ | none | 752 | 558 | - | MD **0.13 points lower** (4.05 lower to 3.79 higher) | ⨁◯◯◯ Very low^a,c,d^ |

**CI:** confidence interval; **MD:** mean difference

#### Explanations

a. Downgraded one level for risk of bias because both contributing trials were judged to have some concerns on RoB 2, mainly related to the randomization process, missing outcome data, and/or selection of the reported result. These limitations could plausibly have affected the estimated treatment effect.

b. Not downgraded for inconsistency. Although statistical heterogeneity was moderate (I² = 57.4% for ADAS-Cog/11 and 59.5% for ADAS-Cog/13), both trials showed effects in the same direction, their confidence intervals overlapped, and the differences in effect magnitude did not materially alter the interpretation. The I² estimates were also based on only two trials.

c. Downgraded two levels for very serious inconsistency because the two trial estimates were in opposite directions and statistical heterogeneity was very high (I² = 89%), with no clear explanation for the discrepancy.

d. Downgraded one level for imprecision because the confidence interval was wide and included potentially meaningful benefit, no effect, and possible harm (MD −0.13, 95% CI −4.05 to 3.79).

**Question: Memantine compared with placebo in patients with vascular dementia**

| **Certainty assessment** | | | | | | | **№ of patients** | | **Effect** | | **Certainty** |
| --- | --- | --- | --- | --- | --- | --- | --- | --- | --- | --- | --- |
| **№ of studies** | **Study design** | **Risk of bias** | **Inconsistency** | **Indirectness** | **Imprecision** | **Other considerations** | **Memantine** | **Placebo** | **Relative (95% CI)** | **Absolute (95% CI)** |  |
| **ADAS-Cog/11 total score (follow-up: 28 weeks; assessed with: Alzheimer's Disease Assessment Scale – Cognitive subscale (11-item version))** | | | | | | | | | | | |
| 2 | randomized trials | serious^a^ | not serious | not serious | not serious | none | 377 | 375 | - | MD **2.2 points lower** (3.24 lower to 1.15 lower) | ⨁⨁⨁◯ Moderate^a^ |
| **GBS total score (follow-up: 28 weeks; assessed with: Gottfries–Bråne–Steen Scale (GBS))** | | | | | | | | | | | |
| 2 | randomized trials | serious^b^ | not serious | not serious | serious^c^ | none | 311 | 284 | - | MD **1.93 points lower** (4.69 lower to 0.84 higher) | ⨁⨁◯◯ Low^b,c^ |

**CI:** confidence interval; **MD:** mean difference

#### Explanations

a. Downgraded one level for risk of bias because both contributing trials were judged to have some concerns on RoB 2, primarily related to missing outcome data and insufficient reporting of the randomization process, which could have affected the estimated treatment effect.

b. Downgraded one level for risk of bias because one contributing trial was judged to be at high risk of bias, mainly owing to concerns regarding selection of the reported result, while the other trial had some concerns.

c. Downgraded one level for imprecision because the confidence interval crossed the line of no effect and included both potential benefit and slight harm (MD −1.93, 95% CI −4.69 to 0.84).

**Question: EGb 761® compared with placebo in patients with vascular dementia**

| **Certainty assessment** | | | | | | | **№ of patients** | | **Effect** | | **Certainty** |
| --- | --- | --- | --- | --- | --- | --- | --- | --- | --- | --- | --- |
| **№ of studies** | **Study design** | **Risk of bias** | **Inconsistency** | **Indirectness** | **Imprecision** | **Other considerations** | **EGb 761®** | **Placebo** | **Relative (95% CI)** | **Absolute (95% CI)** |  |
| **Syndrom-Kurztest total score (follow-up: range 22 weeks to 24 weeks; assessed with: Syndrom-Kurztest total score)** | | | | | | | | | | | |
| 3 | randomized trials | serious^a^ | very serious^b^ | not serious | serious^c^ | none | 151 | 132 | - | MD **2.65 points lower** (5.17 lower to 0.12 lower) | ⨁◯◯◯ Very low^a,b,c^ |

**CI:** confidence interval; **MD:** mean difference

#### Explanations

a. Downgraded one level for risk of bias because one contributing trial was judged to be at high risk of bias, mainly due to deviations from intended interventions and missing outcome data, while the other two trials had some concerns on RoB 2.

b. Downgraded two levels for very serious inconsistency because statistical heterogeneity was extreme (I² = 92.8%), the magnitude of effect varied substantially across trials, and leave-one-out analyses materially changed the pooled estimate and its statistical significance.

c. Downgraded one level for imprecision because the confidence interval was wide and ranged from a very small effect close to no difference to a potentially large benefit, based on only 283 participants.
