# Supplementary material for: Pharmacological interventions targeting cognition and related clinical outcomes in vascular dementia: a systematic review and meta-analysis of randomized controlled trials
Source: Eur J Clin Pharmacol. 2026 Aug 24;82(9):240. doi: 10.1007/s00228-026-04158-9 (PMC13503495; doi:10.1007/s00228-026-04158-9)
Supplement: Supplementary file 2 [file 228_2026_4158_MOESM6_ESM.docx]

| **Summary of Findings: memantine versus placebo for vascular dementia** | | | | | | |  |
| --- | --- | --- | --- | --- | --- | --- | --- |
| Outcome and follow-up | Patients (studies), N | Relative effect (95% CI) | **Absolute effects (95% CI)** | | | Certainty | What happens |
|  |  |  | **Placebo** | **Memantine** | **Difference** |  |  |
| ADAS-Cog/11 total score, Alzheimer's Disease Assessment Scale – Cognitive subscale (11-item version) Follow-up: 28 weeks | 752 (2 RCTs) | - | 2.28 points | 0.08 points | **-2.2** (-3.24 to -1.15) | ⨁⨁⨁◯ Moderate^a^ | Memantine probably results in a slight reduction in ADAS-Cog/11 total score. |
| GBS total score, Gottfries–Bråne–Steen Scale (GBS) Follow-up: 28 weeks | 595 (2 RCTs) | - | 2.9 points | **0.97 points** | **-1.93** (-4.69 to 0.84) | ⨁⨁◯◯ Low^b,c^ | Memantine may result in little to no difference in GBS total score. |
| **CI:** confidence interval; **MD:** mean difference | | | | | | |  |

a. Downgraded one level for risk of bias because both contributing trials were judged to have some concerns on RoB 2, primarily related to missing outcome data and insufficient reporting of the randomization process, which could have affected the estimated treatment effect.

b. Downgraded one level for risk of bias because one contributing trial was judged to be at high risk of bias, mainly owing to concerns regarding selection of the reported result, while the other trial had some concerns.

c. Downgraded one level for imprecision because the confidence interval crossed the line of no effect and included both potential benefit and slight harm (MD −1.93, 95% CI −4.69 to 0.84).

| **Summary of Findings: EGb 761® versus placebo for vascular dementia** | | | | | | |  |
| --- | --- | --- | --- | --- | --- | --- | --- |
| Outcome and follow-up | Patients (studies), N | Relative effect (95% CI) | **Absolute effects (95% CI)** | | | Certainty | What happens |
|  |  |  | **Placebo** | EGb 761® | Difference |  |  |
| Syndrom-Kurztest total score, Syndrom-Kurztest total score Follow-up: range 22 weeks to 24 weeks | 283 (3 RCTs) | - |  | - | **-2.65** (-5.17 to -0.12) | ⨁◯◯◯ Very low^a,b,c^ | EGb 761® may slightly reduce the Syndrom-Kurztest total score compared with placebo, but the certainty of the evidence is very low. |
| **CI:** confidence interval; **MD:** mean difference | | | | | | |  |

a. Downgraded one level for risk of bias because one contributing trial was judged to be at high risk of bias, mainly due to deviations from intended interventions and missing outcome data, while the other two trials had some concerns on RoB 2.

b. Downgraded two levels for very serious inconsistency because statistical heterogeneity was extreme (I² = 92.8%), the magnitude of effect varied substantially across trials, and leave-one-out analyses materially changed the pooled estimate and its statistical significance.

c. Downgraded one level for imprecision because the confidence interval was wide and ranged from a very small effect close to no difference to a potentially large benefit, based on only 283 participants.
