# Supplementary material for: Pharmacological interventions targeting cognition and related clinical outcomes in vascular dementia: a systematic review and meta-analysis of randomized controlled trials
Source: Eur J Clin Pharmacol. 2026 Aug 24;82(9):240. doi: 10.1007/s00228-026-04158-9 (PMC13503495; doi:10.1007/s00228-026-04158-9)
Supplement: Supplementary file 3 [file 228_2026_4158_MOESM7_ESM.docx]

**SWiM reporting checklist**

Checklist for synthesis without meta-analysis,locations refer to the revised manuscript and supplementary materials.

| **SWiM item** | **Reporting requirement** | **Location** |
| --- | --- | --- |
| 1a | Grouping of studies for synthesis | Methods, Sections 3.3-3.4; Supplementary Table 3, Sheets A–G |
| 1b | Rationale for the grouping used | Methods, Sections 3.1 and 3.4 |
| 2 | Standardized metric or transformation | Methods, Section 3.4; original measurement scales retained and effect directions harmonized |
| 3 | Synthesis method | Methods, Section 3.4; structured tabulation and narrative interpretation of magnitude, direction, and precision |
| 4 | Criteria for prioritizing results | Methods, Sections 3.3-3.4 |
| 5 | Investigation of heterogeneity | Methods, Section 3.4; heterogeneity was assessed descriptively through grouping by intervention, comparator, instrument, and follow-up because most non-pooled comparisons were supported by single trials. |
| 6 | Certainty of the evidence | No formal GRADE assessment was conducted for SWiM-only outcomes; confidence was interpreted descriptively using RoB 2, precision, consistency, sample size, and completeness of reporting. |
| 7 | Data presentation | Supplementary Table 3, Sheets A–G; Results, Sections 4.4-4.5 |
| 8 | Reporting results of the synthesis | Results, Sections 4.4-4.5 |
| 9 | Limitations of the synthesis | Discussion, Section 5.3 |

*Source: Campbell M, McKenzie JE, Sowden A, et al. Synthesis without meta-analysis (SWiM) in systematic reviews: reporting guideline. BMJ. 2020;368:l6890.*
