# Supplementary material for: Pharmacological interventions targeting cognition and related clinical outcomes in vascular dementia: a systematic review and meta-analysis of randomized controlled trials
Source: Eur J Clin Pharmacol. 2026 Aug 24;82(9):240. doi: 10.1007/s00228-026-04158-9 (PMC13503495; doi:10.1007/s00228-026-04158-9)
Supplement: Supplementary file 4 [file 228_2026_4158_MOESM8_ESM.docx]

**Comparison with previous systematic reviews and meta-analyses**

Effect estimates are reproduced in the orientation used by each source. For EGb 761®, positive values in Masserini et al. indicate benefit, whereas negative values in the present review indicate improvement because lower SKT scores are better.

| **Review** | **Population and scope** | **Evidence base / method** | **Relevant numerical findings** | **Relationship to the present review** |
| --- | --- | --- | --- | --- |
| Present review | VaD-focused trial populations, predominantly clinically diagnosed VaD or separately extractable VaD subgroups, with one disclosed combined probable-VaD/AD-with-cerebrovascular-disease trial. | 16 parallel-group RCTs; pairwise meta-analysis only for the same intervention, comparator, scale/version, and comparable end-of-treatment assessment; SWiM for other outcomes | Galantamine ADAS-Cog/11: MD -2.01 (-3.18 to -0.85). Memantine ADAS-Cog/11: MD -2.20 (-3.24 to -1.15). EGb 761® SKT: MD -2.65 (-5.17 to -0.12), I²=92.8%. | Incremental contribution is methodological and interpretive: VaD-focused and scale-specific pooling, a broad study-level map of non-poolable cognitive outcomes under SWiM, explicit robustness/certainty assessment, and appraisal of functional and global translation rather than identification of new efficacy signals. |
| Kavirajan & Schneider 2007 [14] | VaD; cholinesterase inhibitors and memantine | 8 placebo-controlled RCTs; fixed-effect drug-specific analyses | Cognitive ADAS effects ranged from MD -1.10 for rivastigmine to -2.17 for donepezil 10 mg. Functional or behavioural benefit was generally absent. | Established the pattern of small cognitive effects with uncertain clinical and functional importance; the present review confirms this pattern under RoB 2/GRADE and stricter scale-specific pooling. |
| Chan et al. 2018 [26] | Traditional Chinese herbal medicines for VaD | 47 trials; 3,581 participants; 18 formulations; international and Chinese databases | No overall herbal-medicine effect was estimated because of substantial clinical and design heterogeneity; certainty ranged from moderate to very low. | Demonstrates that the Chinese-language herbal evidence is much broader than the single Tianzhi trial captured here. The present review is not a comprehensive TCM synthesis. |
| McShane et al. 2019 [13] | Memantine across dementia syndromes; VaD subgroup | 2 mild-to-moderate VaD trials for ADAS-Cog | VaD cognitive function: MD -2.15 (95% CI -3.25 to -1.05); moderate certainty. | Closely matches the present ADAS-Cog/11 estimate, MD -2.20 (95% CI -3.24 to -1.15); therefore confirmatory, not a new efficacy signal. |
| Battle et al. 2021 [12] | VaD and other VCI; cholinesterase inhibitors | 8 RCTs; pairwise and Bayesian network meta-analysis | Galantamine ADAS-Cog: MD -2.01 (95% CI -3.18 to -0.85); moderate certainty; magnitude considered unlikely to be clinically important. | Identical to the present galantamine ADAS-Cog/11 estimate. The present review adds VaD-focused eligibility, separation of scale versions, and integration with functional and SWiM evidence. |
| Masserini et al. 2023 [28] | Broad VCI spectrum; randomized and non-randomized interventions | 118 studies; 19,223 participants; 63 intervention types; 125 outcomes | Descriptive mapping showed marked heterogeneity in populations, interventions, comparators, and outcomes. | The present review uses a narrower clinically diagnosed VaD framework and excludes non-randomized and non-pharmacological evidence. |
| Dang et al. 2024 [27] | VaD pharmacological treatments | 194 RCTs; 21 drugs; Bayesian networks for MMSE, ADL, and adverse reactions | Ranked interventions rather than preserving all instrument-specific effects; ADAS-Cog was not a primary network outcome. | Broader ranking question and larger Chinese-language evidence base. The present review avoids network ranking because the eligible network is sparse and outcomes are non-equivalent. |
| Masserini et al. 2025 [29] | Broad VCI spectrum; pharmacological and non-pharmacological interventions | 173 trials; 22,347 participants; 91 interventions; random- or fixed-effect meta-analysis by intervention | Memantine cognition: Cohen's d 0.33 (95% CI 0.11 to 0.55). Ginkgo SKT: unstandardized improvement +2.56 (95% CI -0.17 to 5.29), very low certainty; n=283. | Memantine is directionally consistent. The Ginkgo magnitude is similar to the present MD -2.65 after scale harmonization, but the confidence interval differs, illustrating analytical fragility. |

**Abbreviations:** ADAS-Cog, Alzheimer's Disease Assessment Scale-Cognitive Subscale; ADL, activities of daily living; CI, confidence interval; MD, mean difference; RCT, randomized controlled trial; SKT, Syndrom-Kurztest; TCM, traditional Chinese medicine; VaD, vascular dementia; VCI, vascular cognitive impairment.
